# Yoga Programme for Type 2 Diabetes Prevention (YOGA-DP) Among High-Risk People in India: A Multicenter Feasibility Randomized Controlled Trial

**DOI:** 10.1007/s13300-023-01395-4

**Published:** 2023-03-31

**Authors:** Kaushik Chattopadhyay, Pallavi Mishra, Kavita Singh, Kalpana Singh, Tess Harris, Mark Hamer, Sheila Margaret Greenfield, Nandi Krishnamurthy Manjunath, Rukamani Nair, Somnath Mukherjee, Nikhil Tandon, Sarah Anne Lewis, Sanjay Kinra, Dorairaj Prabhakaran, YOGA-DP Study Team

**Affiliations:** 1 Lifespan and Population Health Academic Unit, University of Nottingham, Nottingham, UK; 2 Centre for Chronic Disease Control, New Delhi, India; 3 Population Health Research Institute, St. George’s University of London, London, UK; 4 Division of Surgery and Interventional Science, Institute of Sport, Exercise and Health, University College London, London, UK; 5 Institute of Applied Health Research, University of Birmingham, Birmingham, UK; 6 Swami Vivekananda Yoga Anusandhana Samsthana, Bengaluru, India; 7 Bapu Nature Cure Hospital and Yogashram, New Delhi, India; 8 Department of Endocrinology, Metabolism and Diabetes, All India Institute of Medical Sciences, New Delhi, India; 9 Department of Non-communicable Disease Epidemiology, London School of Hygiene and Tropical Medicine, London, UK

**Keywords:** Blood glucose, Prediabetes, Prevention, Yoga, Lifestyle, Physical activity, Diet, Feasibility study, Randomized controlled trial

## Abstract

**Introduction:**

Many Indians are at high risk of type 2 diabetes mellitus (T2DM). The blood glucose level can be improved through a healthy lifestyle (such as physical activity and a healthy diet). Yoga can help in T2DM prevention, being a culturally appropriate approach to improving lifestyle. We developed the Yoga Programme for T2DM Prevention (YOGA-DP), a 24-week structured lifestyle education and exercise (Yoga) program that included 27 group Yoga sessions and self-practice of Yoga at home. In this study, the feasibility of undertaking a definitive randomized controlled trial (RCT) was explored that will evaluate the intervention’s effectiveness among high-risk individuals in India.

**Methods:**

A multicenter, two-arm, parallel-group, feasibility RCT was conducted in India. The outcome assessors and data analysts were blinded. Adults with a fasting blood glucose level of 100–125 mg/dL (i.e., at high risk of T2DM) were eligible. Participants were randomized centrally using a computer-generated randomization schedule. In the intervention group, participants received YOGA-DP. In the control group, participants received enhanced standard care.

**Results:**

In this feasibility trial, the recruitment of participants took 4 months (from May to September 2019). We screened 711 people and assessed 160 for eligibility. Sixty-five participants (33 in the intervention group and 32 in the control group) were randomized, and 57 (88%) participants were followed up for 6 months (32 in the intervention group and 25 in the control group). In the intervention group, the group Yoga sessions were continuously attended by 32 (97%) participants (median (interquartile range, IQR) number of sessions attended = 27 (3)). In the intervention group, Yoga was self-practiced at home by 30 (91%) participants (median (IQR) number of days per week and minutes per day self-practiced = 2 (2) and 35 (15), respectively). In the control group, one (3%) participant attended external Yoga sessions (on Pranayama) for 1 week during the feasibility trial period. There was no serious adverse event.

**Conclusions:**

The participant recruitment and follow-up and adherence to the intervention were promising in this feasibility study. In the control group, the potential contamination was low. Therefore, it should be feasible to undertake a definitive RCT in the future that will evaluate YOGA-DP’s effectiveness among high-risk people in India.

**Feasibility Trial Registration:**

Clinical Trials Registry—India (CTRI) CTRI/2019/05/018893; registered on May 1, 2019.

## Key Summary Points

**Table:** 

Many Indians are at high risk for type 2 diabetes mellitus. The blood glucose level can be improved through a healthy lifestyle (such as physical activity and a healthy diet). Yoga can help in type 2 diabetes mellitus prevention, being a culturally appropriate approach to improving lifestyle.
A multicenter, two-arm, parallel-group, feasibility randomized controlled trial was conducted in India. The outcome assessors and data analysts were blinded. Adults at high risk for type 2 diabetes mellitus were eligible. In the intervention group, participants received a Yoga program to prevent type 2 diabetes mellitus. In the control group, participants received enhanced standard care.
The participant recruitment and follow-up and adherence to the intervention were promising in this feasibility study. In the control group, the potential contamination was low. Therefore, it should be feasible to undertake a definitive randomized controlled trial in the future that will evaluate the effectiveness of the intervention among high-risk individuals in India.

## Introduction

Type 2 diabetes mellitus (T2DM) is a complex metabolic disorder with significant health and socioeconomic consequences, and India has the second-largest T2DM population in the world [1]. An individual at high risk of T2DM has a higher blood glucose level than normal but lower than the level for T2DM, and more than 77 million people in India are at high risk of T2DM [2]. The chances of developing T2DM and its complications are higher among these people compared to individuals with a normal blood glucose level [3]. Indians rapidly develop T2DM from the high-risk state and have one of the highest rates across many ethnicities [4]. The major risk factors for T2DM are physical inactivity and an unhealthy diet, i.e., an unhealthy lifestyle [3]. A cost-effective and sustainable strategy is to screen individuals at high risk of T2DM and provide a lifestyle intervention that is effective [3, 5, 6]. Effective lifestyle interventions can improve their blood glucose level and can have other health benefits [7–9]. However, Indians usually have a low physical activity level, and they usually consume an unhealthy diet [10–12].

Yoga, an ancient mind–body discipline, originated in the Indian subcontinent and incorporates physical activity and a healthy diet [13]. Various styles of Yoga are practiced, but one style is not necessarily superior or more authentic than another, and all focus on the same important topic, i.e., a healthy lifestyle [14]. In general, Yoga’s acceptability is high in India as it fits people’s health beliefs and culture [15, 16]. A gentle approach is used in Yoga, and it is safe and easy to learn, requires minimal guidance and maintenance costs, and can be practiced indoors as well as outdoors [15]. People who are old or with comorbidities can practice it [14, 15]. Yoga includes low-intensity and moderate-intensity practices (< 3.5 kcal/min and 3.5–7.0 kcal/min, respectively) [14, 17–19]. In addition, it is an activity that strengthens the muscles [14]. Therefore, Yoga can contribute to the goal of routine lifestyle advice which is given to individuals at high risk of T2DM to prevent it.

Yoga’s mechanism of action in T2DM and related conditions has been reported before [20]. Briefly, its benefits on T2DM-related risk profiles seem to occur predominantly through the following pathways: (1) by decreasing the activation and reactivity of the sympathoadrenal system and the hypothalamic–pituitary–adrenal axis, and by fostering feelings of well-being, it may lessen the effects of stress and promote multiple beneficial downstream effects on the neuroendocrine status, metabolic function, and related systemic inflammatory responses; (2) by directly stimulating the vagus nerve, it may improve the parasympathetic activity and lead to beneficial changes in the cardiovagal function, energy state, mood, and related neuroendocrine, metabolic, and inflammatory responses. Furthermore, Yoga may reduce body mass index (BMI), and the reduction in BMI lowers the risk of T2DM [3].

Effectiveness systematic reviews suggest the benefits and safety of Yoga in T2DM and related conditions [21–24]. However, the duration of most of the primary studies was short (≤ 3 months), and these studies often had significant methodological limitations. In addition, the intervention development process was not always reported, and some did not describe the intervention in detail. Even if they did, the interventions were heterogeneous. Therefore, robustly designed studies are needed to evaluate the utility of Yoga for preventing T2DM among high-risk individuals. We systematically developed the Yoga Programme for T2DM Prevention (YOGA-DP) [25]. Our aim is to conduct a definitive randomized controlled trial (RCT) in the future that will evaluate YOGA-DP’s effectiveness among high-risk individuals in India compared to enhanced standard care. The chances of successful completion of the definitive RCT will improve if the feasibility of its key elements is tested before commencement [26, 27]. Therefore, the feasibility of undertaking the definitive RCT was explored in this study.

## Methods

The feasibility study protocol is published elsewhere [28].

### Study Design

A multicenter, two-arm, parallel-group, feasibility RCT was conducted. The outcome assessors and data analysts were blinded.

### Study Setting

We conducted this feasibility study at two Yoga centers in India, namely Bapu Nature Cure Hospital and Yogashram (BNCHY, New Delhi) in north India and Swami Vivekananda Yoga Anusandhana Samsthana (S-VYASA, Bengaluru) in south India. These centers are accessed by individuals from a range of socioeconomic backgrounds. We used three languages (English, Hindi, and Kannada) in conducting this feasibility study.

### Sample Size

In a feasibility trial, a formal sample size estimation is not usually needed [29]. It is recommended to recruit at least 50 participants in a feasibility trial [30]. Thus, we recruited a total of 65 participants in this feasibility trial, after taking into consideration the loss to follow-up.

### Screening and Recruitment Strategies

The following strategies were used to inform people about this feasibility study: posters were placed and pamphlets were distributed at several locations (such as in these Yoga centers, health clinics, communities, parks, and religious places) and door-to-door visits were conducted in several communities at different times of the day. Screening camps were organized at several places (such as in these Yoga centers, communities, and religious places) for identifying potential participants. After giving the participant information sheet to potential participants, describing this feasibility study to them, and answering their questions, we requested that those interested in this feasibility trial provide written informed consent. The screening was conducted after receiving written informed consent, i.e., the fasting blood glucose level was assessed using a glucometer (by finger-prick; using either HemoCue Glucose 201^+^ System or Accu-Chek Active) [31, 32]. People with a fasting blood glucose level of 100–125 mg/dL (i.e., potentially at high risk of T2DM) [33] were requested to visit the Yoga center for eligibility assessment, including a confirmatory venous blood test using the standardized glucose oxidase–peroxidase method. This eligibility assessment was conducted after receiving further written informed consent. Blood samples collected were sent immediately to the accredited laboratories for analysis within an hour. If this was not possible for any reason, the serum was immediately separated by centrifugation and stored in a − 80 °C freezer for analysis on the next day.

### Eligibility Criteria

People aged 18–74 years, with a fasting blood glucose level of 100–125 mg/dL (i.e., at high risk of T2DM) [33], and who were safe to do physical activities (checked using the Physical Activity Readiness Questionnaire (PAR-Q)/clinician) [34], willing and able to attend the intervention/control sessions on their own, and able to provide written informed consent were included in this feasibility study. The following people were excluded: pregnant women, those with glycated hemoglobin (HbA1c) ≥ 6.5% (i.e., with T2DM [33]; venous blood test using the high-performance liquid chromatography method) or a serious or uncontrolled medical condition (e.g., cancer), or those who regularly practiced Yoga (i.e., ≥ 150 min/week) or were receiving (or had plans to receive during the feasibility trial period) any related non-pharmaceutical/pharmaceutical intervention (e.g., glucose-lowering medication).

### Randomization

A computer-generated randomization schedule was used to randomize eligible participants to the intervention or control group (1:1, block randomization, stratified by sex and site). Sequential random sampling (140/group) was used, with 35 equal blocks generated using STATA V.15. The block size was four and fixed. This central randomization was performed by an independent statistician, based at the Centre for Chronic Disease Control (CCDC), New Delhi, India, and the allocation was accessed by the recruiting site staff by telephone call. There was an exception to this rule to avoid contamination, i.e., people recruited from the same household or who were close relatives or friends were randomized to the same group. Baseline data were collected after randomization. Participants and intervention/control providers were not blinded to group allocation, but the outcome assessors and data analysts were blinded to the feasibility trial assignment.

### Interventions

#### Intervention (YOGA-DP)

Intervention details are published elsewhere [25]. Briefly, YOGA-DP was a 24-week structured lifestyle education and exercise (Yoga) program and included 27 group Yoga sessions and self-practice of Yoga at home using the program booklet and a video. The intervention was delivered by YOGA-DP instructors, qualified and experienced Yoga teachers with formal training received on the program. The program included Shithilikarana Vyayama (loosening exercises), Surya Namaskar (sun salutation exercises), Asana (Yogic poses), Pranayama (breathing practices), and Dhyana (meditation) and relaxation practices. Female instructors were available for women. Group sessions were delivered locally (e.g., at these Yoga centers and community centers). Participants were able to join at their convenience as these sessions were run at different time points of the day (including evening and weekend sessions). We reimbursed participants’ local travel costs to attend these sessions. We also invited a family member or someone close to the participant to join him/her in these sessions. After completing the program, participants were strongly encouraged to use the intervention materials and maintain a healthy lifestyle in the long term.

We ensured intervention fidelity, and YOGA-DP instructors were regularly trained on the basis of an instructor manual. In addition, we regularly observed and evaluated these sessions using a checklist to ensure delivery according to the manual. Structured and instructive feedback was provided to them to improve their performance.

#### Control (Enhanced Standard Care)

No formal T2DM prevention program is available in India, although some healthcare professionals offer rudimentary advice. Therefore, in the control group, participants received the routine lifestyle advice to prevent T2DM among them in the form of a leaflet, provided by another team member (i.e., different from the YOGA-DP instructor) to avoid contamination.

### Study Parameters and Data Collection

We estimated the essential parameters needed for designing the definitive RCT, e.g., participant recruitment and follow-up (for 6 months), adherence to the intervention, potential contamination in the control group, and standard deviations (SDs) of the outcomes. Details are provided in the feasibility study protocol [28]. Briefly, the following outcomes were assessed: biochemical parameters (fasting blood glucose, HbA1c, total cholesterol, high-density lipoprotein, low-density lipoprotein, very low-density lipoprotein, and triglyceride), physiological parameters (systolic blood pressure, diastolic blood pressure, and heart rate), anthropometric parameters (weight, BMI, and waist circumference), lifestyle (diet, physical activity (the International Physical Activity Questionnaire (IPAQ)-Short was used and categorized into low and moderate/high and vigorous, moderate, walking, and sitting) [35], tobacco usage, and alcohol consumption), health-related quality-of-life (the EuroQol-5D (EQ-5D) dimensions were categorized into no (level 1; no problems) and yes (level 2 to 5; problems)) [36], depression, anxiety, and stress (the Depression, Anxiety and Stress Scale (DASS) dimensions were categorized into normal and mild/moderate/severe/extremely severe) [37], and self-efficacy (for assessing confidence in participant’s ability to practice Yoga) [38].

### Data Analyses and Reporting

For categorical data, numbers and percentages were calculated. For continuous data, summary measures of mean or median and spread were calculated. In terms of the percentage of loss to follow-up and withdrawal, the two groups were compared using the chi-squared test. Being a feasibility trial, it was not powered to find a difference in trial outcomes at 6 months between the two trial arms. However, unadjusted mean difference (MD) or odds ratio (OR) with a 95% confidence interval (CI) were reported to indicate initial estimates of effects. Subsequently, we conducted the analysis of covariance (ANCOVA) for four critical outcomes (fasting blood glucose, HbA1c, BMI, and waist circumference), and regression coefficient and 95% CI were reported. In model 1, the respective baseline value was adjusted for; in model 2, the respective baseline value and age were adjusted for. The analysis was based on the intention-to-treat principle. There was no plan to conduct an interim analysis. STATA V.15 was used to analyze the data. The extension of the Consolidated Standards of Reporting Trials (CONSORT) statement for randomized pilot and feasibility trials was used to report the results [39].

### Ethics and Related Issues

The study was conducted in accordance with the Declaration of Helsinki. The research ethics committees of the following institutes gave ethics approval: Faculty of Medicine and Health Sciences, University of Nottingham (UK), CCDC (India), BNCHY (India), and S-VYASA (India). This feasibility study was performed in accordance with relevant guidelines and regulations. We obtained written informed consent from participants. This feasibility trial was registered with the Clinical Trials Registry—India (CTRI) (CTRI/2019/05/018893; registered on May 1, 2019). India’s Health Ministry’s Screening Committee (HMSC) also approved this feasibility study. The independent Trial Steering Committee (TSC) monitored and supervised this feasibility study.

### Serious Adverse Events

We planned to collect information on serious adverse events (including hospitalization for at least 24 h and mortality) occurring in the feasibility trial participants that might be attributed to the interventions. An independent clinician was to adjudicate the association of any such event to the interventions on the basis of medical and scientific judgment.

### Participant Withdrawal

We planned to withdraw participants from the feasibility trial on the basis of their request or at the discretion of the site investigator, e.g., in case the participant was no longer safe to do physical activities (determined by PAR-Q/clinician) [34] or was diagnosed with diabetes (and would receive the standard treatment).

## Results

### Recruitment and Follow-up

**Fig. 1:**
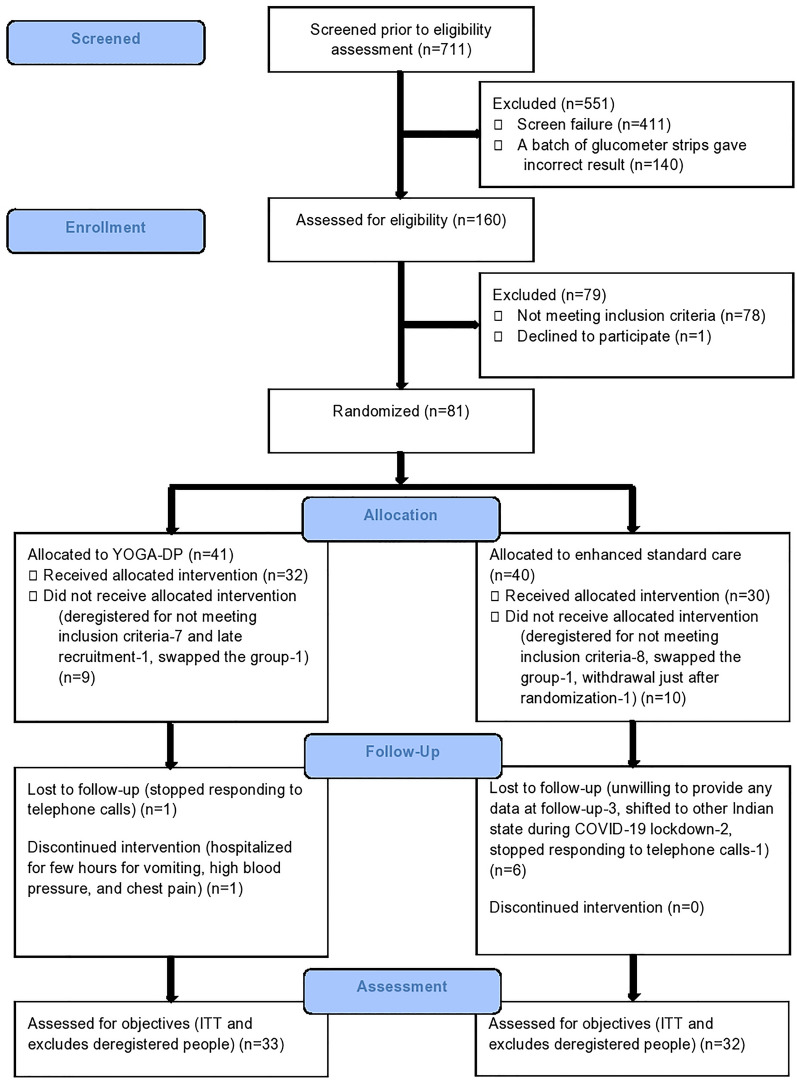
CONSORT flowchart

In this feasibility trial, participants were recruited from 18 May 2019 to 19 September 2019 (i.e., when the first person was approached to participate and the last participant was randomized, respectively). It took 4 months and 2 days to recruit participants. We approached 727 people to participate. Of these, 711 were screened before eligibility assessment and 160 were assessed for eligibility. Sixty-five participants (33 in the intervention group and 32 in the control group) were randomized, and this excludes deregistered participants who did not meet the inclusion criteria but were recruited or who were recruited late. Five participants were randomized to the same group as the first participant (as either they were from the same household or were close relatives or friends). Fifty-seven (88%) participants were followed up for 6 months (32 in the intervention group and 25 in the control group). There was one (3%) loss to follow-up in the intervention group and six (19%) loss to follow-up in the control group, and the difference was statistically significant (*p* = 0.04). There was no withdrawal in the intervention group and one (3%) withdrawal in the control group, but the difference was statistically insignificant (*p* = 0.31) (see Fig. 1 CONSORT flowchart for details). As a result of the COVID-19 lockdown in India, the follow-up of one intervention group participant was delayed and limited information was collected over the telephone from two intervention group participants and one control group participant.

### Intervention Adherence

In this feasibility study, the group Yoga sessions were continuously attended by 32 (97%) participants, and one participant discontinued because of hospitalization for a few hours for vomiting, high blood pressure, and chest pain (this was not counted as a serious adverse event). The median (interquartile range, IQR) number of group Yoga sessions attended was 27 (3). Yoga was self-practiced at home by 30 (91%) participants, and the median (IQR) number of days per week and minutes per day self-practiced was 2 (2) and 35 (15), respectively.

### Potential Contamination in the Control Group

One (3%) participant attended external Yoga sessions (on Pranayama) for 1 week during the feasibility trial period (self-reported). Yoga classes were available outside, but YOGA-DP was not available externally.

### Outcomes

Table 1 reports the baseline characteristics of the feasibility trial participants. The mean (± SD) age of participants was 42.1 (7.7) years, and 25 (38%) were female. The mean (± SD) fasting blood glucose was 110.3 (8.3) mg/dL, HbA1c was 5.9 (0.3) %, BMI was 27.6 (5) kg/m^2^, and waist circumference was 93 (13.2) cm. At baseline, the two groups were mostly similar except for a few variables, which could be due to the small sample size.

**Table 1:** Baseline characteristics of the feasibility trial participants

	YOGA-DP, *N* = 33	Enhanced standard care, *N* = 32
Age (years)	41.3 (7.4)	42.8 (8.0)
Sex		
Male	20/33 (61%)	20/32 (62%)
Female	13/33 (39%)	12/32 (38%)
Mother tongue (language)		
Indo-Aryan	23/33 (70%)	22/32 (69%)
Dravidian	10/33 (30%)	9/32 (28%)
Sino-Tibetan	0/33 (0%)	1/32 (3%)
Married	33/33 (100%)	32/32 (100%)
Formal education of > 10 years	25/33 (76%)	20/32 (62%)
Employed	16/33 (48%)	11/31 (35%)
Gross monthly household income (₹)	26,896.8 (32,303.9)	47,322.6 (50,708.6)
Obesity	6/33 (18%)	4/30 (13%)
Hypertension	2/33 (6%)	1/32 (3%)
Dyslipidemia	0/32 (0%)	1/30 (3%)
Coronary heart disease	0/33 (0%)	0/32 (0%)
Stroke	0/33 (0%)	0/32 (0%)
Peripheral arterial disease	0/33 (0%)	0/32 (0%)
Family history of diabetes	14/31 (45%)	14/32 (44%)
Fasting blood glucose (mg/dL)	110.3 (8.0)	110.4 (8.7)
HbA1c (%)	5.9 (0.3)	5.9 (0.4)
Total cholesterol (mg/dL)	200.7 (43.7)	206.3 (41.0)
High-density lipoprotein (mg/dL)	45.0 (11.9)	45.7 (13.7)
Low-density lipoprotein (mg/dL)	118.9 (29.1)	120.5 (27.2)
Very low-density lipoprotein (mg/dL)	29.5 (18.4)	30.5 (16.5)
Triglyceride (mg/dL)	148.5 (92.2)	140.9 (66.3)
Systolic blood pressure (mmHg)	115.2 (9.8)	117.1 (12.7)
Diastolic blood pressure (mmHg)	74.7 (8.4)	74.2 (8.7)
Heart rate (beats/min)	79.0 (11.8)	81.7 (13.0)
Weight (kg)	69.0 (14.7)	69.8 (11.6)
Height (cm)	160.3 (11.2)	157.4 (8.8)
BMI (kg/m^2^)	26.9 (5.6)	28.2 (4.4)
Waist circumference (cm)	91.1 (15.1)	94.9 (10.8)
Diet		
High-fat/deep-fried food		
No	3/33 (9%)	4/32 (13%)
Occasionally	10/33 (30%)	15/32 (47%)
Regularly (at least 3–4 times/week)	20/33 (61%)	13/32 (40%)
Fruit and vegetables		
< 5 portions/day	27/33 (82%)	27/32 (84%)
≥ 5 portions/day	6/33 (18%)	5/32 (16%)
Physical activity (IPAQ-Short)		
Low	7/26 (27%)	6/22 (27%)
Moderate	19/26 (73%)	16/22 (73%)
High	0/26 (0%)	0/22 (0%)
Vigorous (mins/week)	210.0 (160.6)	210.0 (110.6)
Vigorous (mins/week)^a^	195.0 (75.0–345.0)	255.0 (90.0–300.0)
Moderate (mins/week)	152.0 (205.9)	10.0 (*)
Moderate (mins/week)^a^	52.5 (20.0–300.0)	10.0 (10.0–10.0)
Walking (mins/week)	40.2 (18.9)	40.4 (30.3)
Walking (mins/week)^a^	30.0 (30.0–60.0)	30.0 (20.0–45.0)
Sitting (mins/day)	333.9 (174.3)	339.0 (186.0)
Sitting (mins/day)^a^	360.0 (180.0–480.0)	360.0 (180.0–450.0)
Current tobacco usage	1/33 (3%)	4/32 (12%)
Current alcohol consumption	2/33 (6%)	1/32 (3%)
Health-related quality-of-life (EQ-5D)		
Mobility problems	6/33 (18%)	6/32 (19%)
Problems in taking self-care	2/33 (6%)	2/32 (6%)
Problems in doing usual activities	4/33 (12%)	2/32 (6%)
Pain/discomfort	13/33 (39%)	11/32 (34%)
Anxiety/depression	9/33 (27%)	7/31 (23%)
Visual analog scale score (0 to 100)	69.8 (19.1)	69.0 (16.5)
Depression, anxiety, and stress (DASS)		
Depression^b^	2/33 (6%)	3/32 (9%)
Anxiety^b^	2/33 (6%)	4/32 (12%)
Stress^b^	2/33 (6%)	2/32 (6%)
Self-efficacy in Yoga practice (0 to 100)	71.5 (22.8)	70.3 (20.2)

Values are mean (± SD) or* n*/*N* (%) unless otherwise indicated

*Not enough data to calculate

^a^Values are median (IQR)

^b^Mild/moderate/severe/extremely severe

Table 2 reports the unadjusted outcomes at 6 months.

**Table 2:** Unadjusted outcomes at 6 months

	YOGA-DP, *N* = 33	Enhanced standard care, *N* = 32	Unadjusted MD/OR (95% CI)
Fasting blood glucose (mg/dL)	104.5 (12.0)	107.6 (11.8)	− 3.19 (− 9.60, 3.23)
HbA1c (%)	5.6 (0.4)	5.6 (0.5)	− 0.07 (− 0.32, 0.18)
Total cholesterol (mg/dL)	219.9 (66.7)	207.6 (62.7)	12.32 (− 22.68, 47.33)
High-density lipoprotein (mg/dL)	52.6 (15.4)	49.4 (16.4)	3.27 (− 5.35, 11.89)
Low-density lipoprotein (mg/dL)	139.6 (46.9)	126.0 (40.0)	13.57 (− 10.27, 37.41)
Very low-density lipoprotein (mg/dL)	28.5 (18.3)	27.5 (15.2)	1.00 (− 8.23, 10.23)
Triglyceride (mg/dL)	153.8 (106.0)	139.2 (76.9)	14.58 (− 36.16, 65.32)
Systolic blood pressure (mmHg)	121.4 (11.2)	119.8 (13.4)	1.67 (− 4.82, 8.15)
Diastolic blood pressure (mmHg)	78.2 (7.3)	76.3 (8.6)	1.92 (− 2.25, 6.10)
Heart rate (beats/min)	77.8 (7.1)	79.0 (9.3)	− 1.15 (− 5.47, 3.17)
Weight (kg)	69.1 (14.3)	68.9 (11.2)	− 0.27 (− 6.61, 7.14)
BMI (kg/m^2^)	26.5 (5.0)	28.3 (4.1)	− 1.74 (− 4.19, 0.72)
Waist circumference (cm)	89.6 (15.2)	93.8 (9.7)	− 4.19 (− 11.07, 2.68)
Diet			
High-fat/deep-fried food			
No/occasionally	20/32 (62%)	16/26 (61%)	Ref
Regularly (at least 3–4 times/week)	12/32 (38%)	10/26 (39%)	1.07 (0.36, 3.16)
Fruit and vegetables			
< 5 portions/day	28/32 (88%)	20/26 (77%)	Ref
≥ 5 portions/day	4/32 (12%)	6/26 (23%)	0.45 (0.11, 1.82)
Physical activity (IPAQ-Short)			
Low	5/28 (18%)	0/21 (0%)	
Moderate/high	23/28 (82%)	21/21 (100%)	*
Vigorous (mins/week)	155.0 (204.8)	270.0 (52.0)	− 115 (− 453.6, 223.6)
Vigorous (mins/week)^a^	60.0 (15.0–390.0)	300.0 (210.0–300.0)	–
Moderate (mins/week)	72.5 (112.7)	23.6 (18.4)	48.9 (− 43.6, 141.5)
Moderate (mins/week)^a^	37.5 (30.0–60.0)	15.0 (10.0–30.0)	–
Walking (mins/week)	37.5 (15.8)	48.3 (29.7)	− 10.8 (− 24.5, 3.0)
Walking (mins/week)^a^	30.0 (30.0–60.0)	42.5 (30.0–60.0)	–
Sitting (mins/day)	160.3 (144.4)	242.0 (224.9)	− 81.6 (− 184.9, 21.7)
Sitting (mins/day)^a^	120.0 (30.0–240.0)	120.0 (30.0–420.0)	–
Tobacco usage			
Never/past	32/33 (97%)	28/32 (88%)	Ref
Current	1/33 (3%)	4/32 (12%)	0.22 (0.02, 2.07)
Alcohol consumption			
Never/past	31/33 (94%)	31/32 (97%)	Ref
Current	2/33 (6%)	1/32 (3%)	2.00 (0.17, 23.21)
Health-related quality-of-life (EQ-5D)			
Mobility problems			
No	29/32 (91%)	20/26 (77%)	Ref
Yes	3/32 (9%)	6/26 (23%)	0.41 (0.09, 1.93)
Problems in taking self-care			
No	31/32 (97%)	20/26 (77%)	Ref
Yes	1/32 (3%)	6/26 (23%)	0.13 (0.01, 1.19)
Problems in doing usual activities			
No	27/32 (84%)	20/26 (77%)	Ref
Yes	5/32 (16%)	6/26 (23%)	0.21 (0.04, 1.16)
Pain/discomfort			
No	32/32 (100%)	18/26 (69%)	
Yes	0/32 (0%)	8/26 (31%)	*
Anxiety/depression			
No	32/32 (100%)	21/26 (81%)	
Yes	0/32 (0%)	5/26 (19%)	*
Visual analog scale score (0 to 100)	76.6 (13.1)	71.2 (12.0)	5.43 (− 1.33, 10.18)
Depression, anxiety, and stress (DASS)			
Depression			
Normal	32/32 (100%)	24/26 (92%)	
Mild/moderate/severe/extremely severe	0/32 (0%)	2/26 (8%)	*
Anxiety			
Normal	32/32 (100%)	22/26 (85%)	
Mild/moderate/severe/extremely severe	0/32 (0%)	4/26 (15%)	*
Stress			
Normal	32/32 (100%)	25/26 (96%)	
Mild/moderate/severe/extremely severe	0/32 (0%)	1/26 (4%)	*
Self-efficacy in Yoga practice (0 to 100)	74.1 (15.0)	68.5 (20.7)	4.86 (− 4.64, 14.37)

Values are mean (± SD) or* n*/*N* (%) unless otherwise indicated

^a^Values are median (IQR)

*Not enough data to calculate

Biochemical, physiological, and anthropometric parameters—The fasting blood glucose, HbA1c, weight, BMI, and waist circumference were lower in the intervention group compared to the control, but the differences were statistically insignificant. The high-density lipoprotein was higher in the intervention group compared to the control, but the difference was statistically insignificant.

Lifestyle—The percentage of participants reporting no/occasional intake of high-fat/deep-fried food was higher in the intervention group compared to the control. The moderate physical activity time was higher in the intervention group compared to the control, but the difference was statistically insignificant. Sitting time was lower in the intervention group compared to the control, but the difference was statistically insignificant. The percentage of participants reporting no tobacco usage was higher in the intervention group compared to the control. In addition, the odds of tobacco usage were lower in the intervention group compared to the control, but the difference was statistically insignificant.

Health-related quality-of-life—The percentage of participants reporting no problems in all five dimensions of EQ-5D was higher in the intervention group compared to the control. In addition, the odds of problems in the three dimensions (mobility problems, problems in taking self-care, and problems in doing usual activities) were lower in the intervention group compared to the control, but the differences were statistically insignificant. The visual analog scale score was higher in the intervention group compared to the control, but the difference was statistically insignificant.

Depression, anxiety, and stress—The percentage of participants reporting no problems in all three dimensions of DASS was higher in the intervention group compared to the control.

Self-efficacy in Yoga practice—The self-efficacy was higher in the intervention group compared to the control, but the difference was statistically insignificant.

Table 3 reports the adjusted four critical outcomes at 6 months.

**Table 3:** Adjusted four critical outcomes at 6 months

	YOGA-DP, *N* = 33	Enhanced standard care, *N* = 32	Model 1* Regression coefficient (95% CI)	Model 2** Regression coefficient (95% CI)
Fasting blood glucose (mg/dL)	104.5 (12.0)	107.6 (11.8)	− 3.96 (− 9.81, 1.89)	− 3.79 (− 9.72, 2.13)
HbA1c (%)	5.6 (0.4)	5.6 (0.5)	− 0.07 (− 0.29, 0.16)	− 0.05 (− 0.28, 0.18)
BMI (kg/m^2^)	26.5 (5.0)	28.3 (4.1)	− 0.56 (− 1.00, − 0.11)	− 0.56 (− 1.02, − 0.11)
Waist circumference (cm)	89.6 (15.2)	93.8 (9.7)	− 1.92 (− 4.91, 1.06)	− 1.89 (− 4.90, 1.12)

Values are mean (± SD) unless otherwise indicated

*ANCOVA analysis adjusted for the respective baseline value

**ANCOVA analysis adjusted for the respective baseline value and age

Fasting blood glucose, HbA1c, BMI, and waist circumference—After adjustment for the respective baseline value (model 1) and baseline value and age (model 2), BMI was found to be lower in the intervention group compared to the control, and the difference was statistically significant (regression coefficient − 0.56; 95% CI − 1.00 to − 0.11 and regression coefficient − 0.56; 95% CI − 1.02 to − 0.11, respectively). In addition, fasting blood glucose, HbA1c, and waist circumference were lower, but the differences were statistically insignificant.

### Serious Adverse Events

There was no serious adverse event.

## Discussion

We conducted a multicenter feasibility RCT in India to assess the feasibility of undertaking the definitive RCT in the future that will evaluate YOGA-DP’s effectiveness among high-risk individuals in India. The participant recruitment and follow-up and adherence to the intervention were promising in this feasibility study. There was low potential contamination in the control group. Compared to other T2DM prevention RCTs in India and globally [27, 40], the recruitment in this feasibility trial was without any major hurdles. In this feasibility study, the dropout rate was 12% which is consistent with the findings of a systematic review (less than 15–20% in most RCTs on Yoga interventions) [41]. The only follow-up-related challenge we faced was towards the end of this feasibility trial, and this was due to the COVID-19 lockdown in India. Therefore, it should be feasible to undertake a definitive RCT. This feasibility study provided estimates of important parameters needed for designing the definitive RCT.

In the definitive RCT, the first co-primary outcome will be the incidence of T2DM, indicated by either a fasting blood glucose level of at least 126 mg/dL or an HbA1c level of at least 6.5% at 1-year follow-up [3]. Repeat testing will be conducted in asymptomatic participants to confirm the diagnosis [3], and they will only be included as achieving the co-primary outcome if repeat testing of either fasting blood glucose or HbA1c is above the cutoff. The second co-primary outcome will be BMI at 1-year follow-up [42]. The reduction in BMI lowers the risk of T2DM [3]. It should be noted that compared to other ethnicities, South Asians are at higher risk of T2DM at equivalent BMI levels [43]. We expect that the cumulative incidence of T2DM over 1 year will be 18% in the control group [44, 45]. In the intervention group, we will be interested in a 50% reduction in the cumulative incidence of T2DM [46, 47]. Therefore, in the definitive RCT, we will need to recruit at least 356 participants per group (a total of 712), with 90% power and assuming significance (alpha; two-tailed) of 2.5% to conservatively allow for co-primary outcomes. This sample size will be sufficient for the other primary outcome, considering the mean (± SD) BMI at the baseline was 27.6 (5) kg/m^2^ in this feasibility trial and our interest in at least a 5% reduction in BMI at 1 year in the intervention group [42, 48].

Initial estimates (as this feasibility trial was not powered for effectiveness) show some beneficial effects of the intervention on many critical outcomes, including fasting blood glucose, HbA1c, BMI, and waist circumference. In the adjusted models, BMI was lower in the intervention group compared to the control and the difference was statistically significant. No serious adverse events occurred. Similar beneficial effects and safety of Yoga on T2DM-related outcomes have been synthesized in several effectiveness systematic reviews [21–24]. In the future, the adequately powered definitive RCT will be able to provide a clear answer.

In the definitive RCT, if YOGA-DP is found to be effective, it will be a need-sensitive and evidence-based intervention for preventing T2DM among high-risk individuals, not only in India but also globally. Yoga’s popularity is not restricted to India and other South Asian countries, and it is increasingly becoming popular in other nations [49, 50]. T2DM and its associated costs are global concerns, and a low-cost intervention to prevent T2DM will be of worldwide interest. More evidence-based choices will be available to people for preventing T2DM. The future burden of T2DM (such as clinical, personal, and economic) on patients, families, health systems, and economies will be prevented. The prevention of T2DM may extend to the prevention of its complications. Individuals will become healthier overall, and the intervention may empower people at the same time for managing their health. The intervention has become even more relevant during the COVID-19 pandemic, as it can be delivered online or in-person (outdoors or indoors by following the social distancing rules), and Yoga can be self-practiced at home.

Semi-structured qualitative interviews (as part of the qualitative process evaluation) were conducted with feasibility trial non-participants, participants, YOGA-DP instructors, and staff to explore trial- and intervention-related barriers and facilitators [28, 51, 52]. The findings will inform decisions on modifying the definitive RCT and intervention to further improve participation and adherence. For example, the intervention duration was 6 months in this feasibility trial, and we intend to deliver a year-long intervention in the definitive RCT. The relatively short-term follow-up was another limitation in this feasibility trial, and we intend to do long-term follow-ups (at least for a year) in the definitive RCT. In terms of glycemic control, this feasibility trial gave some hints about the effectiveness of the intervention, and we intend to assess the cumulative incidence of T2DM in the definitive RCT as a co-primary outcome. We reimbursed participants’ local travel costs for attending the Yoga sessions at BNCHY, and at SVYASA, Yoga sessions were run locally and close to their residence. The reimbursement could have boosted participant recruitment and follow-up in this feasibility trial and adherence to the intervention; however, this would not be available in real-world settings.

## Conclusions

The participant recruitment and follow-up and adherence to the intervention were promising in this feasibility study. In the control group, the potential contamination was low. Therefore, it should be feasible to undertake a definitive RCT in the future that will evaluate YOGA-DP’s effectiveness among high-risk individuals in India.
